# Nonseminomatous Extragonadal Germ Cell Tumor Presenting As Early Pericardial Tamponade

**DOI:** 10.7759/cureus.7131

**Published:** 2020-02-28

**Authors:** Taha Ahmed, Talal Ahmad, Samra Haroon Lodhi, Tamoor Ahmed

**Affiliations:** 1 Internal Medicine, Cleveland Clinic Foundation, Cleveland, USA; 2 Internal Medicine, Services Hospital, Lahore, PAK; 3 Internal Medicine, King Edward Medical University/Mayo Hospital, Lahore, PAK

**Keywords:** nonsemonomatous, germ cell tumor, pericardial tamponde

## Abstract

Multiple different types of mediastinal masses may be encountered on imaging techniques in symptomatic and asymptomatic patients. The most frequent mediastinal masses in adults are thymoma, lymphoma, thyroid masses, and germ cell tumors. Potential complications of these masses due to localized invasion include hemoptysis, post-obstructive pneumonia, and superior vena cava syndrome. Pericardial tamponade is usually secondary to pericarditis, trauma, infections, radiation, uremia, vascular diseases, and uremia. However, this report presents a case of a young patient who was found to have a large pericardial effusion and early signs of pericardial tamponade, which have not previously been reported as complications of extragonadal germ cell tumors, to the best of our knowledge.

## Introduction

Male germ cell tumors (GCTs) usually arise from the testis; however, a small subset of them is of extragonadal origin. Extragonadal GCTs are rare and do not have a primary testicular involvement [1]. They are usually found along the midline of the body and are most common in the mediastinum, thymus, retroperitoneal organs, and pineal gland [2]. The exact histogenesis has not been elucidated, but is believed to be secondary to improper migration of germ cells along the urogenital ridge to the gonadal ridges during embryonic development. The clinical symptoms are nonspecific and vary according to the tumor location. We present an interesting case of a 28-year-old male who presented with findings of pericardial effusion and tamponade secondary to an anterior mediastinal GCT compressing the right ventricle.

## Case presentation

**Figure 1:**
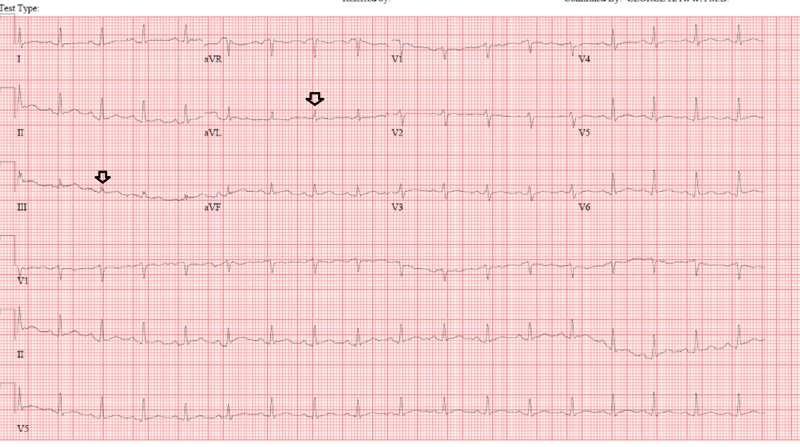
Electrocardiogram showing low voltage QRS complexes

A 28-year-old male presented with a one-week history of pleuritic chest pain, worsening shortness of breath, and non-productive cough. On physical examination, he was in distress and preferred sitting position with his arms elevated to avoid chest pain. Neck exam was positive for jugular venous distention, lungs were clear on auscultation, and cardiac examination showed muffled heart sounds. Vital signs on presentation revealed that he was hypotensive (98/54 mmHg) with a heart rate of 102 beats per minute and afebrile. Electrocardiogram showed sinus tachycardia with low voltage complexes (Figure 1).

**Figure 2:**
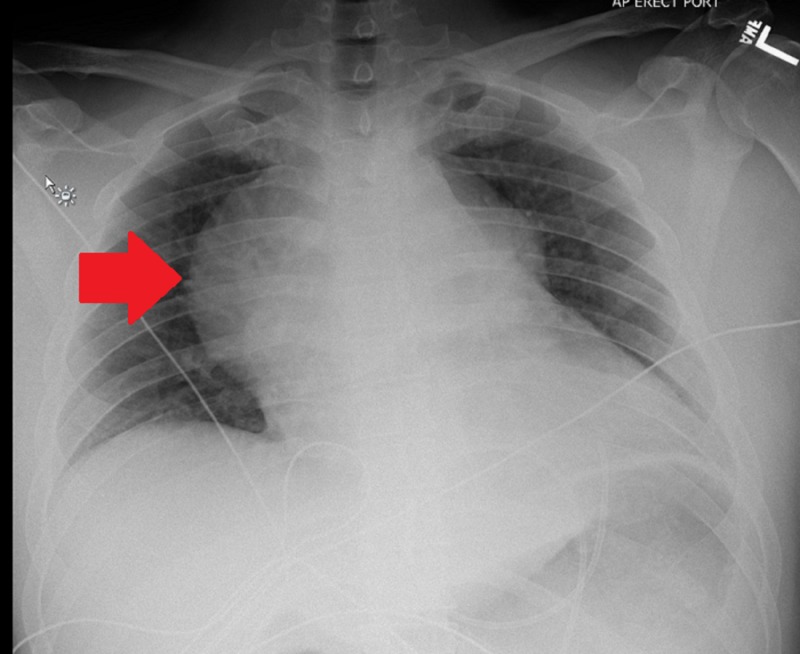
Chest X-ray showing smooth protuberance in the right perihilar region

A chest x-ray showed contour deformity in the right aspect of the mediastinum with a smooth rounded protuberance in the right perihilar region (Figure 2).

**Figure 3:**
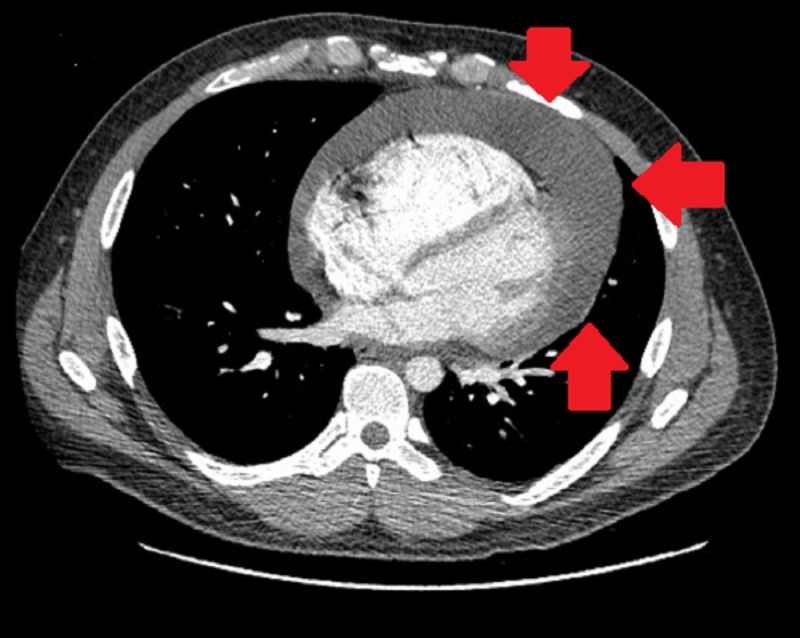
Computed tomography of the chest showing circumferential pericardial effusion and no significant pleural effusion

**Figure 4:**
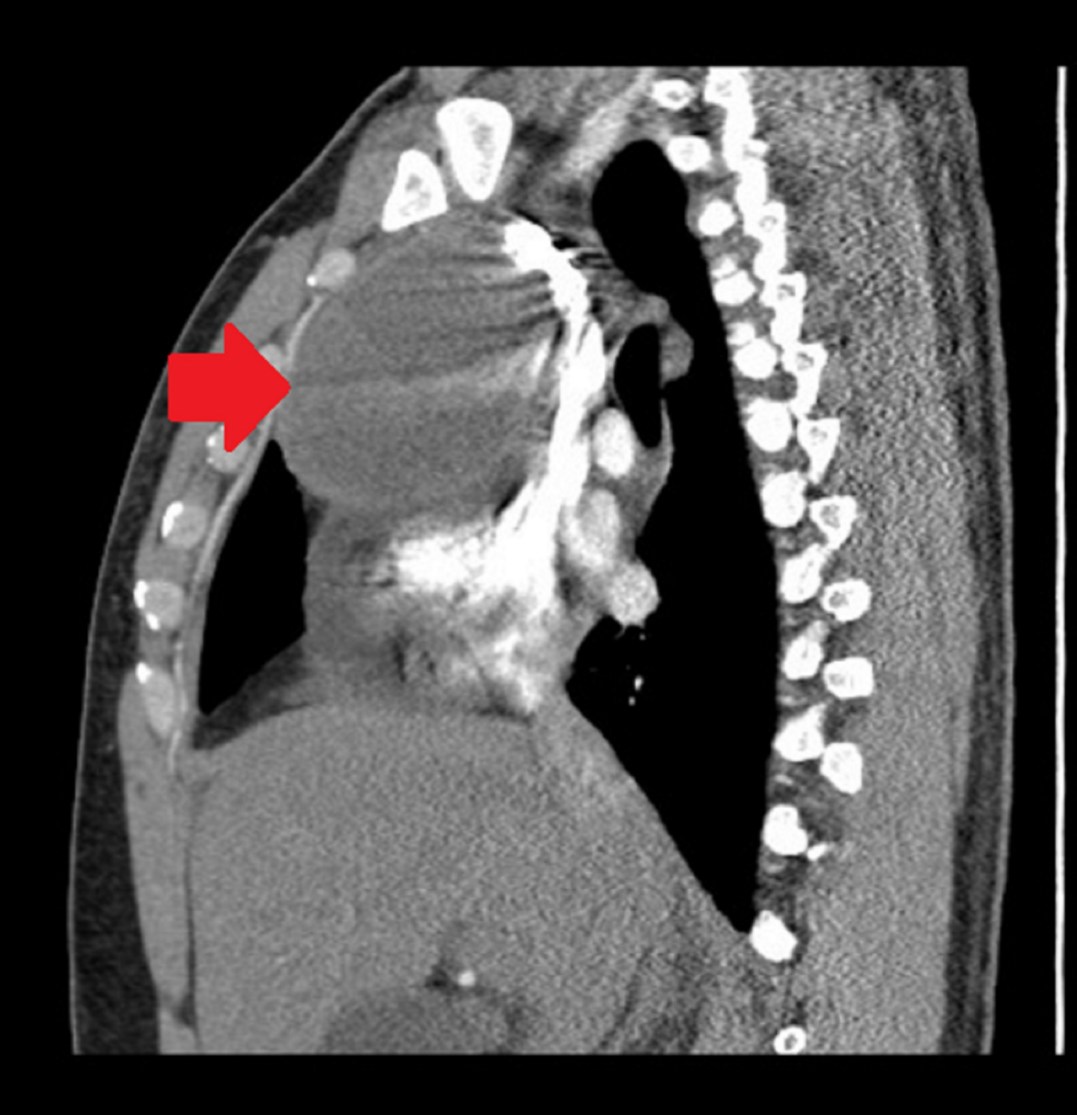
Computed tomography of the chest showing anterior mediastinal mass

D-dimer was elevated, and computed tomography (CT) of the chest was done which, although negative for a pulmonary embolus, revealed a large pericardial effusion and a heterogeneous anterior mediastinal mass (Figures 3, 4).

The patient was admitted to the intensive care unit (ICU) for further management. Bedside echocardiogram in the ICU showed a large circumferential pericardial effusion measuring 4 cm adjacent to both ventricles, inversion of the right atrium and right ventricle, and a large mediastinal mass partially compressing the base of the right ventricle (Video 1).

**Video 1:** Transthoracic echocardiogram showing circumferential pericardial effusion and a large mediastinal mass partially compressing the base of the right ventricle

These findings were suggestive of early pericardial tamponade, and hence it was decided to drain the pericardial effusion percutaneously prior to performing a formal pericardial window. After percutaneous drainage of the pericardial effusion, right thoracoscopy was done with the placement of a pericardial window performed. Cytology samples from pericardial effusion and the pericardium were also sent.

On labs, alpha-fetoprotein (AFP) showed to be very high at 8,132 ng/ml (normal<11 ng/ml), lactate dehydrogenase (LDH) 453U/L (normal=199-220 U/L), and beta-human chorionic gonadotropin (b-HCG) 46 U/L (normal= 4U/L). The pericardial fluid was positive for malignant cells. These results were highly suggestive of an extragonadal GCT, and a tissue biopsy of the mass was taken in the setting of these results. The biopsy results showed yolk sac tumor; CT abdomen and pelvis showed no intra-abdominal pelvic mass or lymphadenopathy.

The findings were discussed with the patient and his family, and they agreed with the initiation of chemotherapy and sperm banking. After sperm banking, the patient was started on platinum-based chemotherapy [3]. The patient got a five-day course of chemotherapy, the chest tube was removed, and he was discharged with a follow-up with the oncologist.

## Discussion

Extragonadal nonseminomatous germ cell tumors (NSGCTs) primarily affect men during the third and fourth decades of life [4]. These tumors are more aggressive and have a worse prognosis than their pure seminoma counterpart [5]. Approximately 85% of patients with mediastinal NSGCTs are symptomatic at the time of diagnosis, with chest pain, hemoptysis, cough, fever, or weight loss being the most common presenting symptoms. Superior vena cava syndrome or pericardial tamponade is occasionally present [6,7]. Our patient presented with cough, shortness of breath, and was in acute distress. He was subsequently found to have a mediastinal yolk sac tumor compressing the heart with pericardial effusion and early signs of cardiac tamponade on bedside echocardiography. Anterior mediastinal NSGCT presenting as early cardiac tamponade is rarely seen in clinical practice, but its early recognition and treatment by emergent pericardiocentesis can be life-saving as was done in our patient [8].

Tumor markers including APF, b-hCG, and LDH help in the diagnosis of these tumors, but the definitive diagnosis and treatment are based on the results of the biopsy of the tumor. Our patient had a high AFP and b-hCG with the tumor biopsy showing a yolk sac tumor. He was admitted under the care of the oncology and thoracic surgical services for emergent chemotherapy; the patient will eventually get four cycles of etoposide, ifosfamide, and cisplatin chemotherapy followed by resection of any residual disease with the goal of treatment as a complete cure [7,9].

## Conclusions

The present case report shows that one of the major causes of anterior mediastinal masses, particularly in young adult males, is an extragonadal GCT. Our patient presented with features of hemodynamic compromise secondary to pericardial tamponade; it was found that an anterior mediastinal tumor was the cause behind it. In order to reach a prompt and accurate diagnosis, a thorough cardiac workup including the use of echocardiogram and CT scans are imminent. Pericardial tamponade as a result of a GCT is rare but if recognized early can be life-saving as demonstrated by our case. The ultimate management of the tumor involves platinum-based chemotherapy followed by complete resection.
